# The Institutional Library

**Published:** 1916-01-08

**Authors:** 


### THE INSTITUTIONAL LIBRARY.

**Diseases of Nutrition and Infant Feeding.** By John Lovett Morse, A.M., M.D., and Fritz B. Talbot, A.M., M.D. (London: Macmillan & Co. 1915. Pp. 346. Price 10s. 6d.)

The scope of this book is chiefly scientific; it is intended to satisfy the demands of students who wish to become acquainted with the original data on which the scientific basis of infant feeding rests. A considerable proportion, however, of the work is devoted to the clinical and practical sides of the subject, including a description of the methods taught in the Harvard Medical School, with which both the authors are connected. In the first section, on physiology and metabolism, an immense amount of bio-chemical research work is sifted, and the references in the literature are given in profusion. The digestive canal of the infant is submitted to a thorough analysis from the chemical, bacteriological, and metabolic aspects. In the second section we have a minute investigation of human milk, with a careful consideration of the numerous factors influencing its composition, and also a study of the clinical considerations and technique of breast feeding. The subject of artificial feeding is naturally considered at great length, and useful tables are given bearing on the chemistry and biology of cow's milk; all the known modifications and manipulations to which milk may be subjected are described in some detail, together with notes on proprietary foods. The practical side of artificial feeding is dealt with at length, and it is worth mentioning that the subject-matter applies equally well to English conditions as to those of the United States, except in a very few minor instances. The prescribing of modified milk is, of course, rather more common in the States than at home, but the authors endeavour to show that the calculation of the formulæ need not be so complicated as it is often considered. Still, even then the amount of arithmetic to be done is no trifle; like everything else, such prescriptions would be little or no trouble if this method were more freely practised. The book finally treats of the digestive disturbances of the infant and some of the commoner diseases of the alimentary canal. Both as a work of reference and as a practical guide this work deserves serious attention.

**Diseases of the Nervous System.** By H. Campbell Thomson, M.D., F.R.C.P. Second Edition. (London: Cassell and Co. Pp. 553. Price 10s. 6d. net.)

Among the smaller text-books of diseases of the nervous system this is quite in the front rank, and it is not surprising that a second edition should have been called for. The arrangement is good, the descriptions are clear, and the style is thoroughly businesslike. For the student commencing the study of the diseases of the nervous system, or the practitioner who requires a concise text-book for an occasional refreshing of the memory, this monograph can be strongly recommended. Within the space at his disposal the author has to be brief, and in regard to treatment he is, perhaps, here and there almost too curt. There are so many nervous disorders for which treatment is almost useless that one could pardon rather more diffuseness in connection with those for which it is of more avail. Dr. Thomson is somewhat non-committal in places. To give an instance, one would like to know his exact views on the best treatment of Graves' disease; they are not apparent in the section devoted to this subject. Perhaps the topic is still so controversial that he prefers to remain rather ambiguous about it. The chapter on tetanus is a little disappointing, in view of the recent increases in knowledge concerning this infection. No mention is made of the premonitory symptoms now recognised; no dosage is given for the antitoxin prophylaxis and treatment, nor any information as to how the serum should be administered. We cannot find any disquisition on so-called "shell shock," one of the war-products which neurologists and many other practitioners see a great deal of nowadays.

**In a French Military Hospital.** By Dorothy Cator. (London: Longmans, Green and Co. 1915. Pp. 99. Price 2s. 6d. net.)

In a general way, and despite exceptions, it is common knowledge in Britain that nursing in France, and indeed in Europe generally, is very far below the standard attained amongst ourselves: this much may be taken as agreed, and not merely as an impression founded on insular conceit. It is not so generally conceded that an equal disparity exists between the medical professions of the two countries; indeed, it would be an untenable proposition to advance. Yet a fairly general agreement is apparent amongst those who have seen French military hospitals at work in this campaign that neither in the nursing nor in the medical and surgical senses are they comparable in efficiency with the corresponding British hospitals. How far the institutional spirit, developed among doctors and nurses alike by the voluntary hospital system as we know it, is responsible for this view, it is difficult to assess at present; we merely record the prevalent opinions of those who have seen these things —opinions which may turn out on fuller investigation to be superficial or erroneous.

In this brightly written book an English lady records her personal experiences and impressions of the particular French military hospital in which she has been working. In so far as she narrates that which she has seen, her testimony agrees with that of other witnesses and confirms the deplorable condition of nursing generally in France: it also, so far as it goes, does not present a very favourable type of French doctor to the reader. The conclusions drawn by the author, when she leaves the realm of experience for that of inference, are much less valuable. Prepossession and prejudice are rife, and we fear that if this volume gains any circulation in France the good effect of the salutary criticisms will be spoilt by the obvious weakness of others.

**With the Turkish Army in the Crimea and Asia Minor.** By T. Buzzard, M.D. (London: John Murray. 1915. Price 10s. 6d. net.)

Among the diminishing band of those who campaigned in the Near East sixty years ago are two of the medical men who went out to be attached to the Ottoman Army as surgeons, and have since attained to eminence in the medical profession. They are Mr. Brudenell Carter, the distinguished ophthalmologist; and the author of this book, Dr. Buzzard. Aided by his diaries and by an excellent memory, he has written an account of his varied experiences which is thoroughly interesting and readable, and contains probably the last first-hand account of Omar Pasha's expedition for the attempted relief of Kars which is ever likely to be published by a living author. Dr. Buzzard appears not to have taken part in any of the historic engagements of the campaign, but he saw many sides of it, and can throw valuable sidelights on some of the minor incidents of the auxiliary operations, as at Kertch, Trebizond, and so forth. It would have been a thousand pities if so much of personal observation and experience should not have been made available for posterity; and if one wonders why Dr. Buzzard has waited so long, at least he may retort with "Better late than never."

**How to Become a Nurse.** Edited by Sir Henry Burdett, K.C.B., K.C.V.O. (London: The Scientific Press, Ltd. Ninth Edition. Price 2s. net.)

This book has now reached its ninth edition, and is so complete with exhaustive information on every point that its practical use for those who wish to enter for nurse training has reached the maximum. Here any woman who wants to take up nursing can learn when and where to begin without adding to the work of busy matrons by writing letters needing answers to several institutions at once, because she is not quite sure where she can best obtain the training she desires.

A clear summary is given of the requirements on the part of each hospital and institution mentioned and of the advantages each offers, as to certificates granted, off-duty times allowed, lectures given, and salaries paid.

Thus, by turning over the leaves, the intending probationer can contrast at a glance the disadvantages and advantages to her own special case of the various training schools. She is in each case given the exact address of the institution to which she decides to apply, and is told whether a personal interview is insisted upon and how long a trial-period is allowed. By reading an introductory chapter she learns how to make her application.

The bulk of the information provided is arranged in the form of an alphabetical list of the names of the various nursing institutions in London and the provinces, and the list includes those for special, as well as for general, training. A further list is also given of those in Scotland, Ireland, the Colonies, and America. These lists are clearly arranged in two divisions, in which institutions with over 100 beds are separated from those with under that number, and the plainly-printed headings make it an easy reference-book to use. In the introductory chapters useful and very practical advice is given as to how a would-be probationer might prepare herself, during an enforced waiting period, for her chosen career, with a sketch of the general requirements of all training schools alike.

No would-be nurse, after studying "How to Become a Nurse," could fail to have a clear and definite idea as to how she can best select a training school and enter upon the course which should make her a capable certificated and fully-trained nurse.

**Women in the Public Health Service.** By Edith Maynard, Memb. Roy. Sanit. Inst. (London: Scientific Press, Ltd. 1915. Pp. 128. Price 1s. 6d.)

In the correspondence columns of ladies' weeklies and nursing journals there appear very frequently queries relating to the ways and means of entry of women into the various branches of the Public Health Service. This shows that women of fair education are appreciating two facts: the one being that the scope of public health work will afford increasing opportunity for the employment of intelligent female inspectors and health visitors; the second fact is that such women are realising the importance, nay, the necessity, of taking steps to make themselves self-supporting in the altered economic conditions of the years to come. The frequency with which inquiries are directed into this channel has created a demand for a source of information more ample than could be expected from editorial answers. This demand, we have pleasure in announcing, has now been met by the publication of a handbook by Miss Edith Maynard, which supplies the requisite information and a good deal besides. Miss Maynard writes with the backing of ample experience in public health work, as she was formerly chief woman inspector in the Public Health Service in Leeds. On the subject of the actual work of an inspector she writes well and to the point; her readers will have no reason to complain of the suppression of the difficulties or unpleasant aspects of the work; but, on the other hand, the true nature of the work, its aims and its results, are set forth with an encouraging clearness. The modern phases of the work, as well as the branches of the older established services, are fittingly described, including factories, outworkers, workshops, schools, inspection of midwives, infant work, and tuberculosis visiting, though the latter is rather briefly dealt with. The second chapter is the one which will appeal to those seeking information regarding the course of training required and the scope of the examinations for various certificates. Much excellent and apposite advice is given under the heading of "Personality," and would-be entrants into the Public Health Service will do well to ponder over this chapter and to ask themselves frankly if they feel certain they possess the most essential of those qualities which will inevitably be demanded of them. A certain amount of knowledge and a certificate are by no means sufficient to ensure a successful or even a smooth and uneventful career. Particulars are given of the various training centres, such as the Royal Sanitary Institute in London, and there is a list of the provincial centres. In the appendix the regulations and syllabus of the examination for Inspectors of Nuisances are set out, and also those of the Sanitary Inspectors' Examination Board; but it is unfortunate that no clear details are given as to the exact scope of any examinations for a health visitor's certificate, a point upon which information is often sought. Something further might have been said, too, on the training to be had for tuberculosis work. These, however, are minor details in a book which will be most helpful to hundreds of women with wide human sympathies who desire to employ their energies upon the improvement of the people and their environment.

**The Secret of Human Power.** By Haydn Burns. (Allen and Unwin, Ltd. Price 5s.)

This is one of those pseudo-scientific books intended for popular consumption which appeared to have considerable vogue before the war, if we may judge from the number of them that were published. The medical author of this work has himself written volumes on "Sleeplessness," "The Secret of Good Health," "Worry," "The Wife: her Book," and so on; a list we mention here as putting into true perspective the inflated opening phrase of the foreword to the present publication, which runs: "This book tells the reader of Pleasure, Pain, Accomplishment, Disappointment, Fate, Failing, Falling Success, Joy, Woe, War, Peace, Survival, Life and Death." It is true that all these big words receive frequent mention, but that Dr. Burns tells the reader anything on these subjects which a sensible person does not know already seems to us extremely improbable. The book's thesis appears to be the power of right thinking, and the value of healthy thought in maintaining a healthy body. In setting it forth a multitude of subjects receive incidental mention, and numerous simple diagrams are employed.

